# Antibacterial and Hydrophobic PLA Biocomposites Enabled by Geraniol-Modified Flax Fibres

**DOI:** 10.3390/polym18020183

**Published:** 2026-01-09

**Authors:** Alona Pawłowska, Magdalena Stepczyńska, Volodymyr Krasinskyi, Joanna Pach

**Affiliations:** 1 Department of Materials Engineering, Kazimierz Wielki University, JK Chodkiewicza 30 St., 85-064 Bydgoszcz, Poland; 2 Lukasiewicz Research Network—Institute for Engineering of Polymer Materials and Dyes, M. Skłodowska-Curie 55 St., 87-100 Toruń, Poland; vkrasinsky82@gmail.com; 3 Department of Lightweight Elements Engineering, Foundry and Automation, Faculty of Mechanical Engineering, Wroclaw University of Science and Technology, Wyb. Wyspiańskiego 27 St., 50-370 Wrocław, Poland; joanna.pach@pwr.edu.pl

**Keywords:** biocomposites, natural fibres, plant fibres modification, natural origin modifiers, plant biocidal compounds, geraniol

## Abstract

In the medical industry, strong disinfectants are used to limit bacterial proliferation on the surface of polymer-based materials; however, they may leave hazardous residues. To prevent potential harm to human health, safer disinfection substitutes are continuously searched. This study evaluates the effect of a natural biocidal modifier, geraniol (GR), on the properties of flax-reinforced biocomposites. Biocomposites containing 80 wt% polylactide (PLA) and 20 wt% flax fibres were prepared, and fibres were modified with 1%, 5%, 10%, or 20% GR. The materials were examined using tensile tests, dynamic mechanical analysis (DMA), differential scanning calorimetry (DSC), thermogravimetry (TG), contact angle measurements, scanning electron microscopy (SEM), and antibacterial activity tests. The incorporation of flax fibres increased the storage modulus from 2730 MPa (PLA) to 3447 MPa, while GR-modified fibres further enhanced stiffness up to 3769 MPa for the 20% GR sample. Strong antibacterial activity against Escherichia coli and Staphylococcus aureus was achieved in biocomposites containing ≥10% GR, with R = 5 and R ≥ 6, respectively. Surface hydrophobicity also improved progressively, and a water contact angle of 92° was obtained at 20% GR. These results demonstrate that geraniol-modified flax fibres effectively impart antibacterial activity and hydrophobicity to PLA biocomposites, indicating their potential for use in sustainable packaging applications and materials for the medical sector.

## 1. Introduction

The problem of residual waste is one of the biggest threats to the current ecological state, and it has already caused irreversible effects. The majority of currently used polymer materials are produced from petrochemical resources and their recycling is difficult. Statistics on polymer waste generation clearly indicate that the packaging industry holds the top position, being responsible for approximately 40% of the total annual waste generated [1]. The waste management issue could be improved by replacing petrochemical polymers with biodegradable polymers in the industries where polymers are commonly used (e.g., medical industry—medical equipment, etc.). For example, polylactide (PLA) is one of the best-known plant-based biodegradable polymers processed from renewable resources. PLA is a thermoplastic material with rigidity and clarity similar to PS or PET and shows high strength and modulus comparable to those of PP and PS [2]. However, biodegradable polymers cause no environmental pollution, unlike petrochemical polymers, because they are biodegradable under industrial composting conditions.

To enhance the mechanical properties of the polymers, different reinforcements are applied. For example, fibres are among the most commonly used reinforcements. The biocomposites that include fibres are more resistant to mechanical stress [3]. A comparison of plant fibres with synthetic ones shows that plant fibres have some undoubted advantages. They are lightweight (unlike glass fibres), cost less, and they are completely biodegradable and processed from renewable resources [4]. Therefore, the development of plant-fibre biocomposites is a huge step for materials engineering with potential applications in everyday life, including food-contact packaging (e.g., trays, disposable cutlery, fresh-produce containers), horticultural items (seedling pots), single-use medical accessories, and touch surfaces in public or clinical environments, where the reduction in microbial load is essential. In such applications, materials must not only exhibit good mechanical performance but also possess inherent biocidal activity, reducing the need for chemical disinfectants [5,6].

Fibre selection was based on their availability in Europe and their physical properties. Flax and hemp are widely cultivated across Europe and represent major sources of natural technical fibres. Comparative studies [7] indicate that flax fibres show greater mechanical characteristics compared with hemp, including higher tensile strength and greater elongation at break.

Besides mechanical strength, materials used in the medical industry must meet other requirements. Due to their close contact with bacteria, materials used in the medical industry (e.g., equipment, elements of public space, etc.) should be pathogen-free. To limit the pathogen proliferation, the densification of the material surfaces is applied. Unfortunately, most disinfectants contain strong chemicals which are harmful to the environment. The toxic residues of these chemicals that remain on the surface have a negative impact on the human health. The application of biocidal modifiers is one of the ways to avoid harmful disinfections. However, most of the currently used biocidal modifiers are also synthetic and hazardous to human health. Therefore, natural and non-toxic modifiers are constantly being sought in both science and industry [6]. Sustainable biocomposites which contain natural biocides are an excellent alternative to petrochemical ones that need to be constantly disinfected with unsafe disinfectants.

Geraniol is a naturally occurring biocidal agent, derived from various plants, including roses, wild roses, geraniums, lavender, lemon, ginger, and orange [8,9,10]. In addition to its strong biocidal properties, geraniol is known for its non-toxic—according to 21 Code of Federal Regulations, Food Drug Administration—antioxidant, and soothing properties, which have made it widely used in the cosmetics industry [11,12,13]. In recent years, geraniol has also gained attention in biomedical systems, including geraniol-loaded nanoparticles for anti-inflammatory or anticancer therapies [14,15] geraniol–cyclodextrin inclusion complexes improving compound stability [16,17], and geraniol-functionalized chitosan derivatives showing promising antimicrobial behaviour suitable for wound-care materials or antimicrobial surface coatings [18]. Geraniol has additionally been incorporated into biopolymer films and hydrogels to enhance antibacterial effectiveness and prolong shelf life in biomedical and food-related applications [19]. However, its interaction with polymer matrices has not been fully explored, making this natural biocidal agent an interesting subject of study.

As well as biocidal activity, hydrophobic properties of the packaging materials are also highly desired. Hydrophilic surfaces are more susceptible to bacterial colonization than hydrophobic ones [20]. The colonization of the surfaces by bacteria leads to a gradual decrease in their properties and, eventually, their biodeterioration (material breakdown). Research shows that the damage caused by microorganisms reduces the lifespan of materials and leads to significant financial losses each year [21].

Flax fibres are plant fibres with a hydrophilic nature. To increase its hydrophobicity, various modification methods are applied [22]. They are often not sustainable and can be harmful for the environment and human health. Additionally, many chemical modifications cause irreversible changes in fibre surface structure, reducing their mechanical properties. Hence, the research focuses on the application of natural, plant-derived modifiers that effectively modify flax fibres and positively affect their surface properties [23]. The introduction of modified fibres into the polymer matrix will contribute to the development of novel biocomposites with biocidal properties and increased hydrophobicity.

Recent studies have extensively explored methods to improve the interface between hydrophilic natural fibres and hydrophobic polymer matrices like PLA. For example, a plasma-enhanced chemical vapour deposition significantly improves the thermo-mechanical properties of flax/PLA composites [24], while their ageing resulted in the deterioration of mechanical properties [25]. There is a growing interest in utilizing natural, bioactive compounds as alternatives to synthetic additives [26]. However, the application of essential oil derivatives, especifically geraniol, as a dual-functional modifier for flax fibres has received limited attention.

## 2. Materials and Methods

### 2.1. Materials

Biocomposite matrix—PLA type 2003D (Cargill Dow LLC, Minnetonka, MN, USA) with a density of ρ = 1.24 g/cm^3^ and a melt flow rate of 4.2 g/10 min (measured at 2.16 kg, 190 °C). The matrix constituted 80% of biocomposite.

Reinforcement—5 mm flax fibres’ length (Ekotex, Namysłów, Poland) which constituted 20% of the biocomposite.

Biocidal modifier—GR C10H18O (Thermo Scientific, Waltham, MA, USA) with a molecular weight of 154.25 g/mol.

Bacterial strains—*E. coli* 8739 and *S. aureus* 6538 P (ATCC, Manassas, VA, USA), needed during the microbiological studies.

### 2.2. Processing Methods

**Figure 1:**
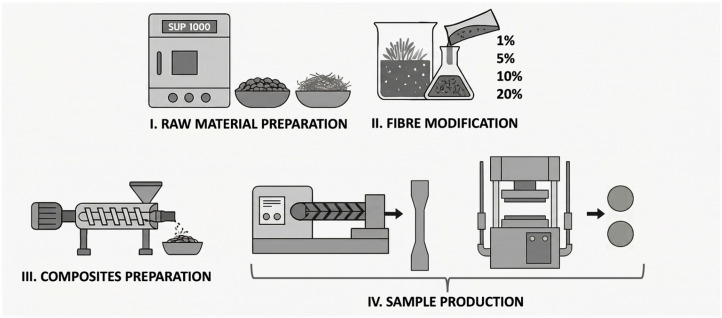
Processing procedure.

Processing was carried out in a five-step procedure (Figure 1). Stages I and II involved the preliminary preparation of the matrix and fibres, while stage III was used to combine these components to form the composite. The final stage (IV) comprised the preparation of two types of samples. A detailed description of each stage is provided in below subsections.

#### 2.2.1. Raw Material Preparation and Fibre Modification

Before processing, all the used materials were dried using laboratory dryer type SUP-100 G (WAMED, Warszawa, Poland). The temperature differed based on specific properties of dried materials: the matrix was dried at 50 °C, while the reinforcement was dried at 60 °C for 12 h. Prepared fibres were modified with GR in concentrations of 1%, 5%, 10%, and 20% (wt%). Table 1 presents the amounts of components contained in the modifying solutions used. The mentioned concentrations were chosen in accordance with our previous research [27] to compare the effects of different modifiers (tannic acid and geraniol) at the same concentrations. The modification technique has been described in detail in [27,28].

#### 2.2.2. Composites Preparation

**Figure 2:**
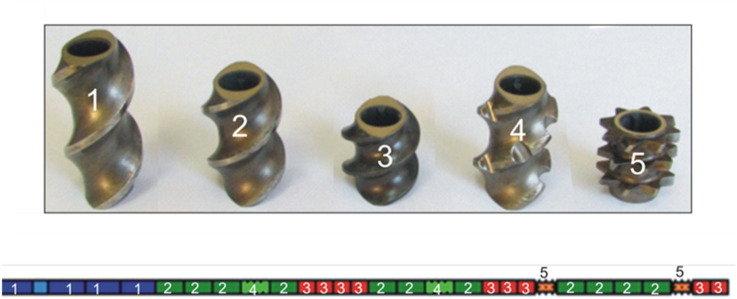
Elements of extruder screw configuration.

Further processing includes thermal methods such extrusion and injection moulding. The co-rotating twin-screw extruder FI = 24 mm, L/D = 40, (Zamak Mercator, Skawina, Poland) ensured better mixing of reinforcement and polymer matrix and improved dispersion of flax fibres in PLA. A special configuration and shape of the screws was applied, intended to minimize the cutting of the fibres into shorter fragments (Figure 2) [28,29]. It did not involve the elements returning and intensively mixing the polymer melt but they were replaced by the elements mainly transporting the melt. The extruder was equipped with a granulator making the extrusion process continuous. The temperature of the extruder head was 185 °C, while the temperatures of the cylinder heating zones (IV–I) were 186 °C, 184 °C, 182 °C, and 180 °C. The speed of the extruder screw was 100 rpm. The polymer matrix was fed at a speed of 133.3 g/5 min, while the reinforcement was fed at a speed of 33.3 g/5 min. The prepared biocomposites contained 80 wt% of the polymer matrix and 20 wt% of the reinforcement.

#### 2.2.3. Sample Production

To produce the paddle-shaped samples needed for mechanical and wettability examinations, an injection moulding machine (TRX 80 ECO 60, Tederic Machinery Manufacture, Hangzhou, China) was used. The process was carried out at 170 °C, 165 °C, 165 °C, 165 °C, and 35 °C assigned to the heating zones (I–III) of the moulding machine cylinder, its head, and mould. Samples were moulded at 24.8 MPa.

A vulcanization press was used to prepare the films needed for the microbiological studies. To prepare 0.5 mm thick samples, 0.7 g of extruded granulate was pressed at 180 °C and 0.7 MPa for 10 s. To prevent deformations, hot films were cooled with compressed air for the next 10 s. Prepared samples were labelled as follows (Table 2).

The samples are indicated as follows: P, N, and MX, where P and N are reference samples, MX indicates samples containing flax fibres that have been modified using geraniol, and X stands for wt%GR.

### 2.3. Examination Methods

#### 2.3.1. Dynamic Mechanical Analysis

The dynamic mechanical analyzer (Q 800, TA Instruments, New Castle, DE, USA) was used to conduct dynamic mechanical analysis (DMA). In this study, the dual cantilever mode was employed to investigate the mechanical properties of the reference sample as well as samples containing non-modified and modified fibres. The experiment was conducted by subjecting bar-shaped samples (35 × 10 × 4 mm) to a consistent frequency of 1 Hz and a deformation amplitude of 15 µm. This evaluation was performed while varying the temperature from 25 °C to 160 °C.

#### 2.3.2. Static Tensile Test

A tensile testing machine (3367 Instron, Norwood, MA, USA) was employed to analyze the tensile strength and Young’s modulus [30,31]. The analysis was carried out following the standard with a strain speed of 20 mm/min. According to ISO 527-2 [31], during the mechanical tests, specimen type 1A (115 × 20/10 × 4 mm) was used. Within this test, twelve samples in the shape of a paddle of each material (reference, containing non-modified and modified fibres) underwent examination. Extreme values recorded during the test were excluded from the analysis.

#### 2.3.3. Thermogravimetric Analysis

Thermogravimetric analysis (TGA) was conducted using a thermogravimetric analyzer (Q500, TA Instruments, New Castle, DE, USA) under a nitrogen atmosphere. A single specimen of each material (reference, containing non-modified and modified fibres) was subjected to examination. The sample masses ranged from 19.7 mg to 20.3 mg. The TGA measurements were conducted over a temperature range from 25 °C to 800 °C with a heating rate of 10 °C/min.

#### 2.3.4. Differential Scanning Calorimetry Analysis

Differential scanning calorimetry (DSC) measurements were conducted within a nitrogen atmosphere using a differential scanning calorimeter (Q200, TA Instruments, USA). Each material (reference, containing non-modified and modified fibres) was studied using a single sample. The sample weights varied from 7.8 mg to 8.2 mg. The measurement temperatures ranged from 20 °C to 210 °C. The DSC curves were captured across three cycles: first heating, cooling, and second heating. A temperature change rate of 10 °C/min was maintained. To ensure the removal of any prior thermal history of the samples, the analysis focused on data derived from the second heating phase. The degree of crystallinity (X_c_) for modified samples was calculated based on total composite mass, including PLA, flax fibres, and geraniol. The below formula was used:

(1) $$X_{c}=\frac{\left({∆H}_{m}-{∆H}_{CC}\right)}{{∆H}_{m100\%}}\cdot 100\%$$

where ΔH_m_ refers to the alteration in the melting enthalpy, ΔH_cc_ to the alteration in cold crystallization enthalpy, and ΔH_m100%_ the to the alteration in melting enthalpy calculated for the pure PLA with maximum X_c_ (for pure 100% crystalline PLA ΔH_m100%_ = 93.6 J/g [32]).

An automated dosing drops system was used during the contact angle measurements, which were performed with a goniometer (DSA100, Krüss GmbH, Hamburg, Germany). During the analysis, water was used as a test liquid for the evaluation of surface wettability of six samples of each biocomposite (reference, containing non-modified and modified fibres). To conduct the studies, drops of the test liquid (with a volume (v) of 7 μL) were placed on the surface of samples at a controlled rate of Δv = 50 μL/min.

#### 2.3.5. Electron Microscope Analysis

Micrographs depicting fractures of the biocomposites after the tensile strength tests were captured using a scanning electron microscope (SEM) (SU8010, Hitachi Ltd., Tokio, Japan). The samples were previously dried at 50 °C using a laboratory dryer type SUP-100 G (WAMED, Warszawa, Poland) to decrease their moisture content, which can deteriorate the quality of the micrographs. To ensure the proper conductivity of tested biocomposites, a thin layer (5 nm) of gold was applied on the surface of the samples using a sputter coater before examination.

#### 2.3.6. Microbiological Testing

The assessment of biocidal properties was carried out following the (“ISO 22196:2024” [33]) standard. According to this standard, the evaluation of biocidal activity was performed against two reference bacterial strains: *E. coli* and *S. aureus.* The strains were cultivated in nutrient broth which served as the growth medium. After the inoculation, the cultures were incubated for 24 h at 37 °C. Subsequently, the optical density assessment method outlined in the standard was used to determine the cell count within a cell suspension. For this purpose, a densitometer (Densitometer II, Pliva-Lachema, Czech Republic) with McFarland’s scale was used [34].

The antibacterial activity (R) is defined as the difference in the logarithm of the number of viable cells found on materials containing biocides (samples containing modified fibres) compared to the reference material (sample containing non-modified fibres). The R coefficient is determined using the following Equation (2):

R = (U_t_ − U_0_) − (W − U_0_) (2)

where U_t_ refers to the average of the common logarithm of the number of viable cells recovered from the reference samples after 24 h, U_0_ refers to the average of the common logarithm of the number of viable cells recovered immediately after inoculation, and W refers to the average of the common logarithm of the number of viable cells recovered from the test samples after 24 h. For each of the tested materials, three tests were performed.

#### 2.3.7. Statistical Analysis

Statistical analysis for mechanical and wettability tests was performed using a one-way analysis of variance (ANOVA) and Tukey’s post hoc test to determine the statistical significance (level α = 0.05) of the effect of fibre and GE content.

## 3. Results and Discussion

### 3.1. DMA

**Figure 3:**
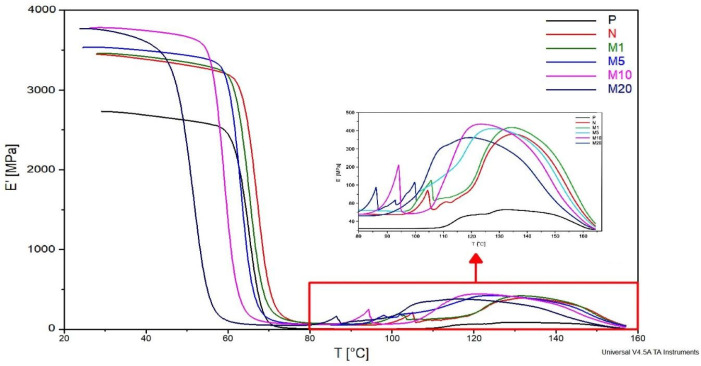
The E′ dependence on the temperature (T) (DMA curve) for the P, N, M1, M5, M10, and M20 samples.

A comparison of DMA curves illustrated in Figure 3 revealed the dependence of storage modulus (E′) on GR concentrations. The introduction of flax fibres into the polymer matrix resulted in an increase in E′ from 2730 MPa (P sample) to 3447 MPa (N sample) at ambient temperature (25 °C). Table 3 sumarizes storage modulus (E′) at ambient temperature. The more than 20% increase in E′ confirms an efficient increase in the biocomposite stiffness after the incorporation of flax fibres into the polymer matrix.

The onset point of glass transition for the N sample is approx. 64 °C. Application of 1% of GR does not affect further stiffness increase—E′ value was 3459 MPa which is similar to E′ of N sample. However, the glass transition of the M1 sample was initiated at a slightly lower temperature (about 62 °C). GR is known for its plasticizing properties [19], which are confirmed by the shifting of the glass transition range for M1. The higher increase in E′ (3533 MPa) was noted for the M5 sample. Also, the beginning of the glass transition was initiated at approx. 60 °C. The same tendencies have been noted in the case of M10 where E′ was approx. 3781 MPa and glass transition started at near 56 °C. The improvement of E′ by more than 300 MPa (compared to the N sample) could be explained by the higher amount of GR, which contains more alkyl chains. The mentioned chains are known for their hydrophobic properties which improve the internal hydrophobicity of biocomposite, resulting in higher E′. A double increase in modifier concentration (M20) caused the glass transition to start at about 47 °C. The increase in GR concentration led to a greater mobility of polymer molecules, which enhances the plasticization effect. In summary, the increase in bending resistance for the M5 and M10 samples in the glassy state temperature range could be explained by alkyl groups contained in GR, which improved the stiffness of the biocomposites. It can be concluded that the most positive effect of modification on the mechanical properties of biocomposites in the glassy state temperature range was achieved in the case of the M10 sample, where E′ was more than 300 MPa higher compared to E′ of the N sample.

A zoomed-in fragment of the curves (Figure 3) in the cold crystallization range (from 80 °C to 150 °C) shows one-step crystallization for the P sample and two-step crystallization for fibre-reinforced samples (both modified and non-modified). The cold crystallization peak of the P sample occurs at around 110 °C, which indicates typical recrystallization for PLA. The first step of the fibre-reinforced sample crystallization occurs as a single peak with a rapid increase in E′. These changes can be explained by the plasticization of the polymer and the rapid increase mobility of chains in both modified and non-modified samples. The mobility of chains in the N sample with a peak at 105 °C could be caused by the melting of fats and waxes which naturally present in flax fibres [35]. A similar peak in the cold crystallization area for PLA reinforced with 20% flax fibres was obtained by Aliotta et al. [36]. The introduction of modified fibres increases the mobility of polymer chains which was confirmed by the shifting of peaks to the lower temperatures (Table 4). These peaks appear at approx. 102 °C, 98 °C, 94 °C and 86 °C for M1, M5, M10 and M20, respectively. It can be concluded that the degree of plasticization increases with the increase of the concentration of the modifier.

It is known that fibres improve the degree of matrix crystallization due to their nucleating properties. The increase in E′ after the previously discussed peaks proves the nucleating nature of flax fibres. The E′ increase for the M1 sample overlaps with that of the N sample. The increase for the M5, M10, and M20 samples begins at lower temperatures. This shift confirms the plasticizing effect of the modifier which intensifies with increasing modifier concentration.

**Figure 4:**
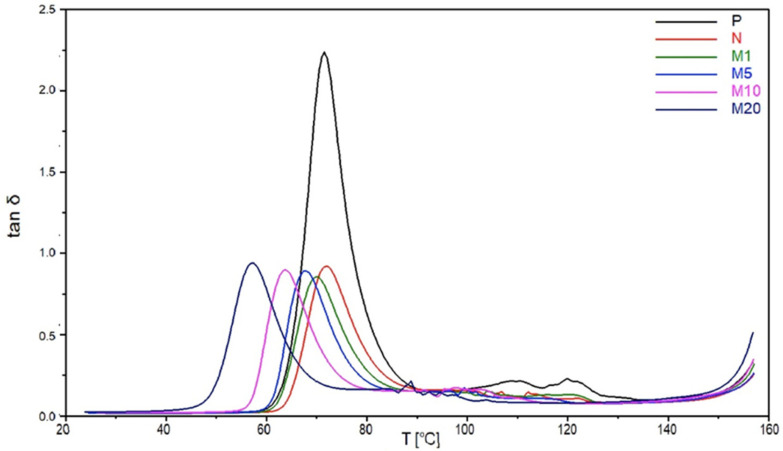
The damping coefficient (tan δ) dependence on the T for the P, N, M1, M5, M10, and M20 samples.

The damping coefficient (tan δ) can indicate the mechanical energy macromolecular segments absorb in their translational movements. Figure 4 illustrates the decrease in tan δ values of samples N, M1, M5, M10, and M20 compared to the tan δ value of the P sample. This change confirms the increased stiffness of the biocomposites due to the presence of flax fibres [37]. The tan δ peak is associated with the glass transition (T_g_) temperature range, which is shifted towards lower temperatures for all of the modified samples [38]. It can be observed that T_g_ temperature decreases with the increase in modifier concentration, because geraniol increases the mobility of polymer chains, resulting in reduced intermolecular cohesion and stress. So, at low modifier levels, we have higher stiffness. This observation confirms the enhancement of the plasticization effect with an increasing modifier concentration.

### 3.2. Static Tensile Test

**Figure 5:**
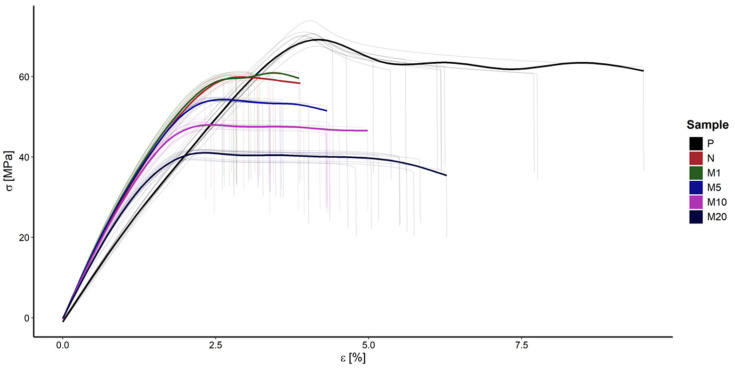
Stress–strain curves for the P, N, M1, M5, M10, and M20 samples. Grey lines represent raw data while bold lines are obtained by averaging the measurements of the same material.

The results obtained during the tensile strength examination, conducted under normal temperature conditions [39], are presented in Table 5. Graphical interpretation of obtained results is showed in Figure 5. As expected, the highest tensile strength (σ_m_) value was noted for the P sample. Examination revealed the decrease in σ after the introduction of flax fibres which confirms an increase in stiffness of the N sample noted in the DMA section. The gradual decrease in σ occurred with an increase in modifier concentration. The σ_m_ values for the N and M1 samples are similar, which indicates an insignificant effect of such a concentration of modifier. However, further increases in modifier concentration leads to a greater decrease in σ. This decrease could be caused due to the deterioration in durability of M5, M10, and M20 samples and their low resistance to deformation. Initially, it was considered that the applied modifier might migrate into the polymer matrix, and terpenoid esters in GR could create a plasticization effect by weakening polymer chain interactions [40,41]. However, analysis of mechanical properties (increase in Young’s modulus and decrease in elongation at break) suggests that the modifier acts as a stiffening agent rather than a classic plasticizer.

The observed reduction in elongation at break (ε_b_) for non-modified and modified samples compared to the P sample indicates increased brittleness of the N sample. The modifier slightly promotes the decrease in ε_b_ values; although the decrease is less than 1 percentage point, due to low baseline values (~4%), this corresponds to approximately a 25% relative reduction in elongation, which is significant. Similar observations have been made in previous studies concerning the effect of tannic acid used as a flax fibre modifier [27].

Young’s modulus (E) value increased by more than 1 GPa after the introduction of fibre, which indicates an improvement in biocomposite stiffness. An increase in Young’s modulus with a simultaneous decrease in tensile strength means an increase in the stiffness of the material, but also its greater susceptibility to brittle fracture. The application of the modifier in concentrations of 1% and 5% slightly enhanced stiffness. However, this effect was disturbed at higher modifier concentrations (M10 and M20). The high amounts of plant agents in biocomposites led to a decrease in polymer chain interactions and deterioration of mechanical properties [42]. In summary, while the application of 1% and 5% modifier concentration improved biocomposite stiffness, it also led to deterioration in resistance to deformation.

The statistical analysis reveals consistent trends between σ_m_, ε_b_, and E across the material variants. For all three mechanical properties tested, σ_m_, ε_b_, and E, the *p* value was <0.001 (statistically significant differences). The results allow us to reject the null hypothesis of equality of means in all groups. To identify specific pairs of groups that differed significantly, Tukey’s test was performed at a significance level of α = 0.05. No significant differences were observed for the following: N vs. M1 in tensile strength (*p* = 0.430); M10 vs. M20 in elongation at break (*p* = 0.149); and M1 vs. M5 (0.479), M5 vs. M10 (*p* = 0.803) in Young’s modulus.

Because GE modification causes significant changes in properties, with higher concentrations (20%) additionally worsening the strength and stiffness compared to 10% modification, a direct comparison of M10 vs. M20 was performed (using Welch’s *t*-test). The statistical analysis reveals that an increase in GE concentration from 10% to 20% significantly reduces σ_m_ by 14.3% (*p* < 0.001), does not significantly affect ε_b_ (*p* = 0.103), and significantly reduces Young’s modulus by 6.3% (*p* < 0.001).

### 3.3. TG

**Figure 6:**
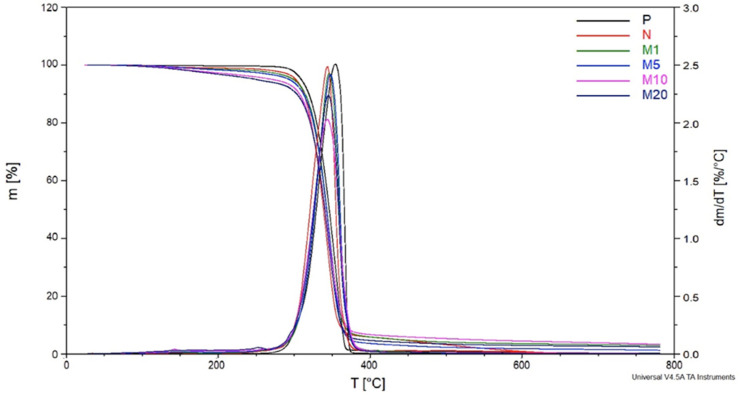
TG and DTG curves for P, N, M1, M5, M10, and M20 samples.

Figure 6 illustrates mass loss (m) in the function of temperature (T) known as the TG curve for both modified, non-modified, and neat samples. Moreover, its first derivative (DTG curve) is also shown. The initial mass loss of fibre-reinforced samples (both modified and non-modified) has been noted before 100 °C, which corresponds to the evaporation of water contained in flax fibres as well as in GR. The P sample began to rapidly lose weight at approx. 300 °C, indicating a decrease in its thermal stability. Similar behaviour from 200 °C to about 300 °C was observed for the N, M1, and M5. The modifier in concentrations of 1% and 5% had an insignificant impact on the thermal properties of biocomposites. However, the mass loss rate in this temperature range was higher for samples containing 10% and 20% of modifier (M10 and M20). This can be explained by higher amounts of GR which undergoes a two-step thermal transition: a) evaporation up to 230 °C, and b) decomposition between 230 °C and 291 °C [16].

Table 6 summarizes mass loss and degradation temperatures for each sample. A slight decrease in the 5% mass loss temperature (T5%) of the N sample compared to the P sample is attributed to the presence of flax fibres which are susceptible to thermal degradation. A further decrease in T5% for modified samples compared to the N sample indicates the presence of GR. The modifier loses its thermal stability after it exceeds 200 °C. The results show that T5% decreased with the increase in modifier concentration. Comparison of M5, M10, and M20 samples revealed the most significant differences in T5%. The T5% of M10 decreased by more than 22 °C compared to the M5 sample. The doubling of the modifier concentration in the M20 sample led to a further decrease in T5% by another 22 °C.

The comparison of 50% mass loss temperatures (T50%) between the N sample and P sample shows similar tendencies as in T5%. Additionally, a decrease of 11 °C was noted in the decomposition temperature (Td). Insignificant changes in T50% and Td were noted for all of the modified samples. The increase in T50% and Td for M1 and M5 compared to the N sample was noted. However, decreased T50% and Td temperatures were noted within increased modifier concentration (10% and 20%). Lower Td values affected by GR in high concentrations were also noted by [18].

Changes in 95% mass loss temperature (T95%) values deviate from the observed dependency. Changes in the temperatures associated with 95% mass loss (T95%) do not align with the expected pattern. The unusually high T95% values can likely be attributed to two main phenomena: char formation (carbonization) and crosslinking, along with thermal stabilization due to high geraniol (GR) content. These factors can lead to rare but extreme temperature peaks. In conditions with limited oxygen, incomplete combustion may result in the formation of carbonaceous char. This char layer acts as a thermal insulator, potentially creating localized hotspots where temperatures can rise significantly above the average. Geraniol contains reactive functional groups that can undergo thermal crosslinking with PLA or its degradation products. At higher concentrations of GR, a crosslinked, thermally stable network may form. This network resists melting and instead tends to undergo slow degradation or carbonization, which can further promote high local temperatures. The combination of slowed decomposition and heat retention contributes to the extreme temperatures recorded in the T95% values. Nevertheless, it is recommended to conduct more detailed studies to determine the reason for such behaviour. Based on the observations, it can be concluded, that GR in low concentrations (1%, 5%) slightly inhibits the degradation rate of biocomposite. Oppositely, high amounts of GR (10% and 20%), which presumably migrated into the polymer matrix, resulted in the deterioration of the thermal stability of M10 and M20. This could be caused by the high amount of hydroxyl group contained in GR, accelerating the degradation process [14,19,43,44]. However, the biocomposites are designed for application within specific temperature ranges, ensuring the thermal stability of their components, including GR. This precautionary measure aims to prevent decomposition or any undesirable reactions.

### 3.4. DSC

**Figure 7:**
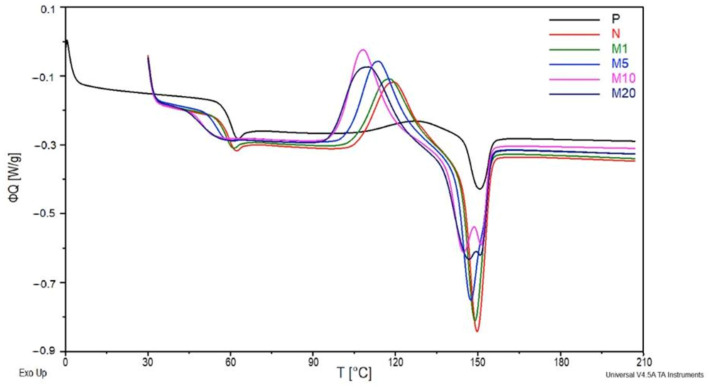
The dependence of ΦQ on T for P, N, M1, M5, M10, and M20 samples.

The changes in heat flow (ΦQ) in the function of temperature are demonstrated in Figure 7. The first endothermic peak assigned to the N sample slightly shifts towards lower temperatures compared to the P sample. It could be caused by the waxes contained in the flax fibres, which act as natural plasticizers in polymer matrix. A similar tendency was noted for the M1 sample, where the mentioned peak is quite similar to the ones of the N sample. With the increase in GR amount, a decrease in the energy needed for the initiation of glass transition was observed. The M5, M10, and M20 samples have no characteristic endothermic peak, their curves started to shift in the endothermic direction with the further soft transition into the baseline. Additionally, the glass transition temperature (T_g_) (Table 7) gradually decreased with the increase in GR concentration. These changes are characteristic for plasticizers, which act as lubricants in polymers [37]. These observations confirm our previous assumptions concerning the plasticizing properties of the applied modifier.

The cold crystallization enthalpy increased after the introduction of flax fibres into the polymer matrix (N sample). Flax fibres are known for their nucleating properties, which increase the PLA crystallization rate (Table 7). Further increases in cold crystallization enthalpy (ΔH_cc_) of samples containing GR are caused by improved chain mobility of plasticized PLA [45]. The highest ΔH_cc_ value was observed in the case of the M20 sample. The 20% GR concentration has the strongest plasticizing effect. The application of non-modified flax fibres led to a shift in the exothermal peak (cold crystallization peak) towards lower temperatures. The decrease in the maximum temperature of the cold crystallization peak (T_cc_) was also noted for all the modified samples. This observation is consistent with previously discussed results concerning the plasticizing properties of GR.

The same trends for ΔH_m_ and Tm were observed. Analysis of the second endothermic peaks (melting peaks) revealed ordinary one-stage melting in the case of P, N, M1 and M5. However, the samples containing 10% and 20% of GR concentration melted in two stages. The volatilization of GR begins at approx. 150 °C, which corresponds to the melting peak. Hence, it can be deduced, that the first stage of melting is associated with the melting of the crystalline phase created by nucleates contained in flax fibres, while the second stage correspondes to the volatilization of the modifier. Presumably, due to the low amounts of modifier in the samples M1 and M5, the two melting stages overlapped into one. In addition to the enhanced crystallization rate with an increase in GR concentrations, the X_c_ degree of crystallinity decreased. This could be attributed to defects in PLA structure caused by GR [45]. Therefore, the deterioration of the mechanical properties of samples containing modified fibres is justified.

### 3.5. Wettability

Table 8 presents the average contact angle ($\bar{\Theta }$_w_) values. The introduction of flax fibres into the polymer matrix does not signifficantly affect the wettability of the biocomposites. However, a slight improvement in $\bar{\Theta }$_w_ was noted after the modification of flax fibres. Low amounts of modifier contained in M1 and M5 samples slightly increased the $\bar{\Theta }$_w_ values. The application of a 10% modifier concentration increased the $\bar{\Theta }$_w_ value by almost 8° compared to N. Materials used in the packaging industry are expected to be hydrophobic due to close contact with food. A material can be considered hydrophobic if its $\bar{\Theta }$_w_ value is greater than 90° [27,46,47].

**Figure 8:**
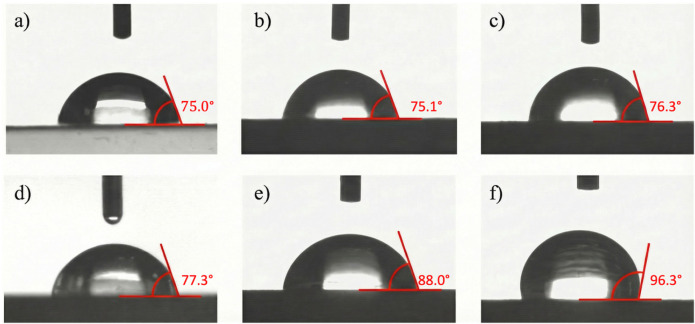
The drops of water placed on the surface of (**a**) P, (**b**) N, (**c**) M1, (**d**) M5, (**e**) M10, and (**f**) M20 samples.

The observed effect was achived after modifying the flax fibres with 20% GR. The $\bar{\Theta }$_w_ value of the M20 sample exceeded 90°, making it hydrophobic. The gradual decrease in the wettability of the biocomposites, noted after modification, could be attributed to the migration of GR into the polymer matrix. The 20% modifier concentration, which contains a high amount of hydrophobic alkyl chains, created a natural hydrophobic barrier on the surface of the M20 sample. Figure 8 illustrates the changes in drop shape with increasing modifier concentration. It can be seen that the shape of the M20 sample significantly differs from that of the other samples.

The ANOVA test showed statistically significant differences between the tested samples (*p* < 0.001). Post hoc analysis (Tukey’s HSD test) revealed no significant differences between samples P, N, M1, and M5 (all pairwise comparisons yielded *p* > 0.05). GE modification at low concentrations (1% and 5%) does not significantly affect the surface wetting properties. On the other hand, the GE modification of flax fibres at concentrations of 10% and 20% results in a statistically significant increase in the hydrophobicity of the biocomposites, with the strongest effect observed at the 20% concentration. Statistical analysis of M10 vs. M20 showed that increasing the geraniol concentration from 10% to 20% leads to a highly statistically significant (*p* = 0.0003) increase in the hydrophobicity of the biocomposite surface.

### 3.6. SEM

**Figure 9:**
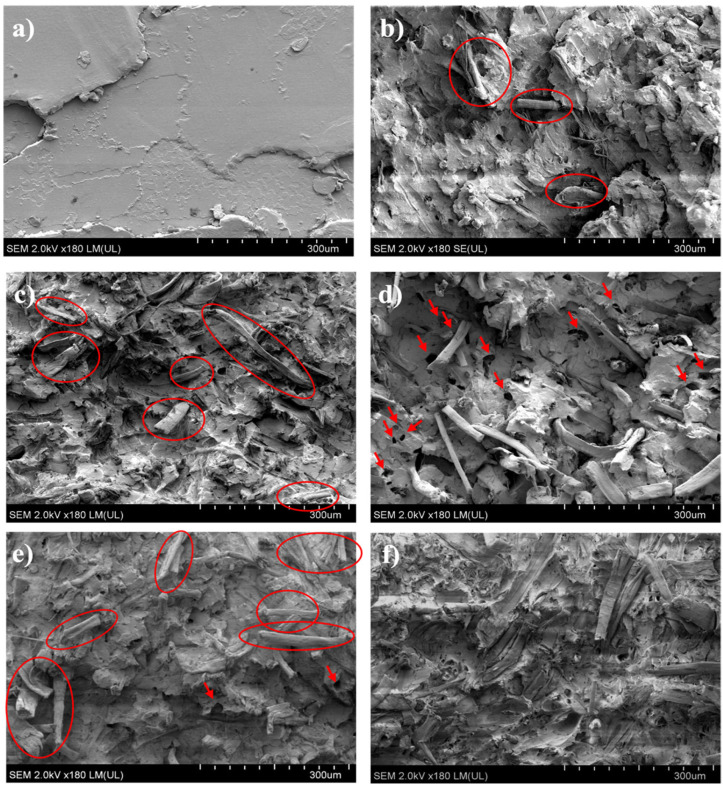
The SEM micrographs of the (**a**) P, (**b**) N, (**c**) M1, (**d**) M5, (**e**) M10, and (**f**) M20 samples. Prostruded fibres highlighted with red circles. Holes in materials indicated with red arrows.

The micrograph shown in Figure 9a presents the P sample with a characteristic layered fracture, whereas the micrograph of the N sample fracture shows a lower number of protruding fibres compared to modified samples. This observation indicates better adhesion between the matrix and non-modified fibres than in the case of modified ones. The greater interfacial adhesion resulted in better transfer of tensile loads from the matrix to the fibres. A similar structure was observed in the M1 sample, where 1% GR concentration did not affect the fibre–matrix interaction (Figure 9c). Protruded fibres were highlighted with circles, while holes with arrows.

The introduction of fibres modified with 5% of the modifier led to the deterioration of interfacial adhesion in M5. The holes seen in Figure 9d remained after the fibres were pulled out. A further increase in modifier concentration (10% and 20%) resulted in a much more debonding structure of M10 and M20 (Figure 9e,f). Along with the structure changes, the number of pulled-out fibres increased. A number of protruded fibres visible in Figure 9f is big, hence no highlights were added. These observations suggest the migration of GR into the polymer matrix, affecting the interfacial adhesion of the biocomposite.

### 3.7. The Biocidal Properties

The biocidal activity of the samples was determined against two bacterial strains—*E. coli* and *S. aureus*. A material is considered biocidal if the R-value is >2 (Table 9). No biocidal activity was observed in the P and N samples. In the case of the M1 sample, the R-value was 0 against *E. coli* and 1 against *S. aureus*. *S. aureus* is a Gram-positive bacterium with no outer membrane, making it more susceptible to modifier penetration. However, the antibacterial effect of GR was noted only at a 5% modifier concentration. It was effective against *E. coli* but not against *S. aureus*, suggesting lower efficiency of low amounts of GR against Gram-positive bacterial strains. To confirm the mentioned assumption, more detailed research is needed. Tests on samples with higher modifier concentrations (M10 and M20) revealed strong biocidal activity against both bacterial strains. In summary, GR exhibited biocidal activity against *E. coli* in the M5, M10, and M20 samples, and against *S. aureus* in the M10 and M20 samples. Determining the biocidal activity of a material is essential for predicting which microorganisms it can effectively combat and what potential applications it may have. Because of its non-toxic nature and enhanced hydrophobicity, the biocomposite is suitable for use in food-contact applications, such as food packaging or disposable packaging.

## 4. Conclusions

This study was carried out to investigate the use of natural, plant-derived modifiers that effectively modify flax fibres and positively affect their surface properties. The introduction of modified fibres into the polymer matrix contributed to the development of novel biocomposites with biocidal properties and increased hydrophobicity. The key findings are as follows:

Mechanical and thermal properties: While low concentrations of geraniol (1–5%) slightly improved stiffness (Young’s modulus), higher concentrations acted as plasticizers, lowering the glass transition temperature (T_g_) and reducing tensile strength. Thermal analysis (TG/DTG) indicated that high geraniol concentrations (≥10%) accelerate thermal degradation.

Antibacterial efficacy: The modification imparted significant biocidal activity to the biocomposites. Samples with 10% and 20% geraniol content achieved a coefficient reduction in R ≥ 5 against *E. coli* and *S. aureus*, effectively inhibiting bacterial proliferation.

Hydrophobicity: A transition from hydrophilic to hydrophobic surface properties was observed. The water contact angle increased progressively with modifier content, exceeding 90° for the composite containing 20% geraniol.

Based on these outcomes, future research will focus on assessing the long-term stability of these biocomposites. Specifically, the plan is to investigate the influence of ageing on selected properties of biocomposites containing geraniol.

## Figures and Tables

**Table 1:** Compositions of different GR solutions.

Solution [1%]	Water [mL]	GR [mL]
1	1782	18
5	1710	90
10	1620	180
20	1440	360

**Table 2:** The description of the sample labels.

Sample Label	PLA Content [%]	Flax Fibres Content [%]	GR Solution Used [%]
P	100	0	0
N	80	20	0
M1	80	20	1
M5	80	20	5
M10	80	20	10
M20	80	20	20

**Table 3:** Storage modulus (E′) at ambient temperature.

Sample	E′ [MPa] at 25 °C
P	2730
N	3447
M1	3456
M5	3369
M10	3568
M20	3769

**Table 4:** The glass transition (T_g_) temperature and damping coefficient (tan δ) peak temperature for P, N, M1, M5, M10, and M20 samples.

Sample	T_g_ [°C]	tan δ
P	71.34	2.2585
N	71.96	0.9239
M1	70.15	0.8626
M5	62.27	0.9097
M10	60.79	0.8951
M20	57.13	0.9431

**Table 5:** The tensile strength studies results obtained for P, N, M1, M5, M10, and M20 samples.

Sample	σ_m_ [MPa]	ε_b_ [%]	E [GPa]
P	69.99 ± 0.73	4.07 ± 0.07	2.25 ± 0.05
N	59.86 ± 0.74	2.91 ± 0.12	3.34 ± 0.10
M1	60.33 ± 0.70	2.77 ± 0.12	3.49 ± 0.14
M5	54.33 ± 0.32	2.51 ± 0.10	3.45 ± 0.04
M10	47.93 ± 0.58	2.32 ± 0.09	3.31 ± 0.15
M20	41.07 ± 0.84	2.25 ± 0.11	3.10 ± 0.07

**Table 6:** Results obtained during the thermogravimetric studies for P, N, M1, M5, M10, and M20 samples.

Sample	T_5%_ [°C]	T_50%_ [°C]	T_95%_ [°C]	T_d_ [°C]
P	309.33	346.10	365.09	354.01
N	302.16	338.58	434.52	343.47
M1	299.16	342.94	435.05	345.87
M5	293.74	342.63	372.85	347.58
M10	271.72	340.61	527.89	343.50
M20	250.36	340.20	384.87	344.82

**Table 7:** Results obtained during DSC studies for P, N, M1, M5, M10, and M20 samples.

Sample	T_g_ [°C]	ΔH_cc_ [J/g]	T_cc_ [°C]	ΔH_m_ [J/g]	T_m_ [°C]	X_c_ [%]
P	60.43	5.86	127.49	6.22	150.70	0.38
N	59.71	20.38	119.52	21.48	149.69	1.18
M1	58.60	20.53	117.96	20.82	148.92	0.31
M5	55.02	22.59	113.85	22.77	147.41	0.19
M10	49.95	22.69	108.35	23.27	144.82	0.62
M20	48.96	24.99	110.26	25.29	146.77	0.32

**Table 8:** The results were obtained during the wettability studies for samples P, N, M1, M5, M10, and M20.

	P	N	M1	M5	M10	M20
$\bar{\Theta }$_w_ [°]	75.0	75.1	76.3	77.3	83.0	96.3

**Table 9:** Biocidal activity of P, N, M1, M5, M10, and M20 against *E. coli* and *S. aureus*.

Sample	*E. coli* [CFU/mL]		*S. aureus* [CFU/mL]	
	T_1_	R	T_1_	R
P	1.5 × 10^7^	0	2.0 × 10^7^	0
N	1.5 × 10^7^	0	1.5 × 10^7^	0
M1	1.5 × 10^6^	0	2.0 × 10^6^	1
M5	2.0 × 10^5^	2	3.0 × 10^6^	1
M10	1.5 × 10^2^	5	1.5 × 10^2^	5
M20	≤1.0 × 10^1^	≥6	1.2 × 10^1^	6

T_0_—the numbers of cells of the tested strains (1.5 × 10^8^ jtk); T_1_—number of bacterial cells after contact time. The time of the contact of bacteria with the tested foil was 24 h.

## Data Availability

The original contributions presented in this study are included in the article. Further inquiries can be directed to the corresponding author.
